# Phylogenetic biodiversity assessment based on systematic nomenclature

**Published:** 2007-02-21

**Authors:** Ross H Crozier, Lisa J Dunnett, Paul-Michael Agapow

**Affiliations:** 1 School of Tropical Biology, James Cook University, Townsville, Queensland, Australia; 2 School of Information Technology, James Cook University, Townsville, Queensland, Australia; 3 VieDigitale Ltd, 20 Matthias Ct., 119 Church Road, Richmond, United Kingdom

**Keywords:** Evolutionary history, phylogenetic diversity, genetic diversity, biodiversity, phylogeny, systematic nomenclature

## Abstract

Biodiversity assessment demands objective measures, because ultimately conservation decisions must prioritize the use of limited resources for preserving taxa. The most general framework for the objective assessment of conservation worth are those that assess evolutionary distinctiveness, e.g. Genetic (Crozier 1992) and Phylogenetic Diversity (Faith 1992), and Evolutionary History (Nee & May 1997). These measures all attempt to assess the conservation worth of any scheme based on how much of the encompassing phylogeny of organisms is preserved. However, their general applicability is limited by the small proportion of taxa that have been reliably placed in a phylogeny. Given that phylogenizaton of many interesting taxa or important is unlikely to occur soon, we present a framework for using taxonomy as a reasonable surrogate for phylogeny. Combining this framework with exhaustive searches for combinations of sites containing maximal diversity, we provide a proof-of-concept for assessing conservation schemes for systematized but un-phylogenised taxa spread over a series of sites. This is illustrated with data from four studies, on North Queensland flightless insects (Yeates et al. 2002), ants from a Florida Transect (Lubertazzi & Tschinkel 2003), New England bog ants (Gotelli & Ellison 2002) and a simulated distribution of the known New Zealand Lepidosauria (Daugherty et al. 1994). The results support this approach, indicating that species, genus and site numbers predict evolutionary history, to a degree depending on the size of the data set.

## Introduction

There is an instinctive and natural desire to preserve all species across the world, but in reality this “Noah’s Ark” approach (Mann & Plummer 1995) is impractical. Resources - financial and otherwise - are limited, the scale of the problem too vast (Agapow et al. 2004), and blanket protection policies are unlikely to be politically successful. Conservation is necessarily a question of economics and prioritization. How can time and money be spent most efficiently? Which species and populations should be targeted for preservation? What metrics can be used for measuring a species importance?

Given the variety of organisms, sites and environments under consideration, it is initially unclear what quality should be measured by any metric of “conservation worth”. Many taxa have qualities that demand their preservation (e.g., being sources of valuable products or other economic benefits, scientific importance, or cultural value), but for the great majority their values are not so clear and are difficult to compare. Even when taxa are clearly “valuable”, questions of priority will arise, because the preservation of one taxon may conflict with that of another. Political success for any conservation scheme is more likely if the proposal is backed by objective measurable data.

Objective criteria for the selection of sites and populations necessary to preserve single species chosen for conservation are relatively well developed (Frankham et al. 2002). But authoritative estimates of the number of species in the world are around 10 million (May 1992, Magurran 2005), so that it is clear that broad-scale solutions are needed rather than dealing with one species at a time.

Ecologists typically regard species richness, the number of species in sites being considered for preservation, as the currency of conservation (Justus & Sarkar 2002, Gaston & Spicer 2004). The consideration of species numbers alone may, however, be insufficient, because of such factors as general imperfect taxonomic knowledge and variation in the level of this knowledge from one group to another. For some time therefore it has been suggested that the phylogenetic distinctiveness of species be taken into account (reviewed by Crozier (1997) and by Mace et al. (2003)), a point of view elegantly encapsulated by Wilson (1992) when he defined biodiversity as the information content in the world’s genomes. The sense of this view is illustrated by the east African great lakes. Some of these are home to more than 1,000 species of cichlid fishes which appear to have evolved over a very short evolutionary time (Meyer 1993). Naively relying on species number alone would value this group more than the ungulates, primates and carnivores combined. Using an approach that weights species by their evolutionary distinctiveness returns the cichlids to a value that intuitively seems more correct and undistorted by “cheap” species.

Early applications of phylogeny to conservation relied purely on topology (reviewed by Crozier (1997)), but the much greater information content in branch-length metrics (recall the cichlid example above) led to their more widespread use and development (Crozier 1992, Faith 1992). Two dimensions can be discerned. One distinction is between measures that consider only the tree connecting the species of interest, as against measures that include the root of the tree connecting the species studied to the rest of life. The other considers the lengths of evolutionary branches (e.g., number of substitutions), as against taking account of saturation of differences (e.g., number of positions with different nucleotides). For example, as two DNA sequences diverge following speciation or gene duplication, differences will accumulate as substitutions occur. With time, substitutions will tend to occur at the same positions as earlier ones, so that the rate of divergence slows even though the rate of evolution does not, a distinction well brought out by the phrase of DeSalle et al. (1987) that eventually Hawaiian Drosophila cease to diverge even while continuing to evolve rapidly. Naturally, saturation occurs for more than DNA sequences–birds and fruit flies continue to evolve, but few would think that they are still becoming more different from each other. “Phylogenetic diversity” (PD) measures retained diversity as the length of tree retained between the group of interest without taking saturation into account (Faith 1992):

(1) $$PD=\sum_{k=1}^{2n-3}d_{k},$$

where *n* is the number of species and d_k,_ is the length of branch k in the tree.

“Genetic diversity” (GD) resembles PD but takes saturation into account (Crozier 1992). Specifically, GD estimates the probability that the set of taxa preserves more than one allele per site:

(2) $$GD=1-\prod_{k=1}^{2n-3}\left(1-p_{k}\right)$$

where p_k_ is the proportion of sites different in state at the two ends of branch, hence 0 ≤ p_k_ ≤ 1. For molecular data, d_k_ is derived from p_k_ according to one or other of the models of sequence evolution.

“Evolutionary history” (EH) is similar to PD but includes the connection of the subtree to the rest of life (Nee & May 1997), by always including the node at the root. For symmetry we define a measure “Genetic history” (GH) which uses (2) above but always includes the root node in calculations, thus resembling EH. PD and GD thus deal with unrooted trees whereas EH and GD require rooted ones. Evolutionary history is attractive compared to PD because the analysis then preserves the context within the rest of life, and is appropriate for this study because of the non-molecular nature of the data.

It has been a truism that conservation of habitats, with thousands or more species each, is preferable to concentrating on conserving particular species, necessarily small in number. The phylogenetic approach goes further, and asks about the evolutionary distinctiveness of species to be conserved. Phylogenetic methods involving whole communities have been applied to aquatic eukaryotic microbes using denaturing gradient gel electrophoresis of total extracted environmental DNA (van Hannen et al. 1998) and to subterranean bacteria via 16S rDNA sequences (Crozier et al. 1999).

There is, however, a major impediment to a more general application of phylogenetic methods to conservation, and that is that the vast majority of groups lack complete phylogenies and this situation is unlikely to be corrected in the near future. A workaround for this problem already exists but has yet to be applied to conservation biology problems.

Systematists generally try to make the arrangement of species into taxa mirror the topology of an inferred evolutionary tree, and the various classificatory levels similarly reflect the systematist’s judgement as to the degree of difference. Thus, surrogate phylogenies can be inferred from systematic nomenclature, and these phylogenies applied in biodiversity assessment. We here illustrate this method and, using species by site (location) data from four other studies, demonstrate its application using multi-platform computer programs.

Estimates of confidence in biodiversity estimates are desirable when they can be made (Crozier 1997). Where surveys are not claimed to yield complete data, the survey data could be used to estimate statistical sufficiency, such as by using bootstrap or jackknife methods to derive confidence limits for EH, PD, GD or GH, and sample coverage methods (Chao & Lee 1992, Chao 2004) to obtain confidence limits for species richness. The entities used for such estimates will differ between groups. For example for social insects the correct unit is closer to the number of colonies (Wilson 1963, Pamilo & Crozier 1997, Chapman & Bourke 2001) because these better approximate the number of reproductives than does the number of sterile or infertile workers. In turn, the number of colonies of a species is approximated by the number of pitfall traps with its workers, rather than the absolute number of workers. Such measures are available in one of the programs discussed here, MeSA, and we discuss their use below.

## Methods

**Figure 1:**
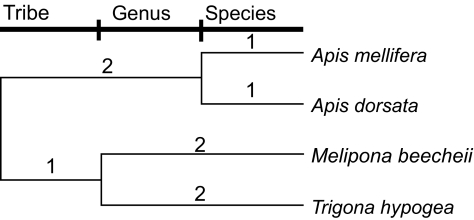
Phylogeny of some social bees inferred from the systematic nomenclature shown in Table 1.

Systematic nomenclature is used to infer a phylogeny of the species under consideration. A branch of equal length is allowed for each level in the hierarchy. An example is shown for a selection of social bees with the systematic nomenclature shown in Table 1, yielding the phylogeny of Figure 1.

The program TreeMaker allows the conversion of systematic nomenclature into an inferred phylogeny (or the importing of an actual phylogeny, if known) and the recording of the presence of the various species across collection sites, either as presence or absence or as abundance data. Branch lengths can either be one for each change of systematic level, or the distance from the root of the tree to the tips can be divided equally. Biodiversity for different combinations of sites is then determined by the species and resultant phylogeny that would be preserved if the sites are retained, according to whichever metric (e.g. PD, GD, GH or EH) is used. The absolute value of the preserved biodiversity varies with the metric used, but the ranks of combinations of sites are the same (Krajewski 1994) and there is for any particular data set (e.g., that of Crozier et al. (1999)) a simple interconversion between PD and GD unique to that data set. The absolute values can be important in intuitive evaluations - for example EH will tend to indicate that more biodiversity is preserved than does PD for the same data.

We have used EH in our calculations here. For the set of bees, a set with *Apis mellifera* and *A. dorsata* preserved will have an EH of 4 and one which also preserves *Melipona beechei* one of 7 (the PD values of these sets are 2 and 7).

The biodiversity preserved by conserving a set of sites is the EH of the species preserved. The program MeSA allows an exhaustive search of combinations of sites, calculating the species richness and EH (and other measures if desired, such as various estimates of species diversity and complementarity) of each combination. Confidence limits for species richness are asymmetric ones obtained via sample coverage methods. For example the estimator Chao84 (Chao 1984) uses information on the abundance of species which are rare but present to estimate the number of species which are rare but absent. Confidence limits for the diversity measure used in an analysis (e.g., EH) are obtained by standard jackknife and bootstrap methods, namely by subsampling from the observations seen in a combination and determining EH for each subsample (see Sokal and Rohlf (1995) for a review). Our implementation of jack-knifing followed standard practice, with each observation being omitted in turn to create a sub-sample.

The algorithm for converting systematic nomenclature into an inferred phylogeny is implemented in two freely available programs, both called TreeMaker. The first is a Java program storing its data in an SQL database, and is available from http://homes.jcu.edu.au/~jc125033/Treemaker.htm. The second, available in Windows and Macintosh versions, stores its data in a structured format in files and is available from http://www.agapow.net/software/treemaker. MeSA is available from http://www.agapow.net/software/mesa.

We used four data sets to explore the properties of our approach. The first of these example data sets contains information on the presence or absence of 273 species of flightless insects in 86 genera from 14 North Queensland localities resulting from a long-running Queensland Museum study directed by G. E. Monteith (Yeates et al. 2002). The tree inferred from systematics is given in the Appendix as a NEXUS file readable by TREEVIEW X. The second data set comes from a transect surveying the occurrences of northern Florida ants in a longleaf pine habitat, involving 72 species in 24 genera from 12 sites (Lubertazzi & Tschinkel 2003). The third data set stems from a study of New England bog ants (Gotelli & Ellison 2002) using an updated data set recording abundances of 34 species at 22 localities. The fourth data set was inspired by the discovery of a second species of the genus *Sphenodon*, which as the sister group to all other lepidosaurs is highly isolated phylogenetically (Daugherty et al. 1990, May 1990). *Sphenodon* is now largely limited to sites lacking introduced rats, with the rate of loss dependent on the particular invasive rat species (C. E. Daugherty, pers. comm.), rendering problematic any examination of the impact of *Sphenodon* on the conservation worth of sites. We therefore used the list of New Zealand lepidosaurs (*Sphenodon* and lizards) given by Daugherty et al. (1994), comprising 62 species placed in five genera, and simulated a set of 15 sites. Each species occurs three times and these occurrences were distributed at random to the 15 sites. The phylogenetic trees and occurrences at sites for the four data sets are given in NEXUS files in the Appendix.

For each dataset, all possible combinations of included sites were generated. From the resultant ensemble of sites the genera, species and EH preserved were calculated. These analyses were performed by MeSA. Including the set of all sites, there are 16,383 combinations for the North Queensland Flightless Insects (NQFI) data, 4,095 for the Florida ants (FLA) data, 4,194,303 for the New England bog ants (NEBA) data and 32,767 for the New Zealand Lepidosauria (NZL) data. All the NQFI and FLA data can be meaningfully graphed, but it was necessary to sample from the NEBA and NZL results to yield a more tractable number of points, chosen to be 20,000.

In order to investigate the effects on EH of phylogenetically divergent species, for each data set we distinguished between site combination-shaving remarkably divergent taxa and those without. The impact of a species on EH is expected to reflect the length of the branch connecting it to the rest of the tree (Crozier 1992, Faith 1992). For the NQFI data we selected *Austrovelia queenslandica* (abbreviated *Austrovelia AV01* in the NEXUS file), the sole member of the Mesoveliidae in this data set, for FLA we selected *Myrmecina americana*, sole representative of its tribe, for the NEBA data we selected *Amblyopone pallipes*, sole member of its subfamily in this ant data set, and for NZL we selected the genus *Sphenodon*.

To illustrate the use of confidence limit calculations, we used the Chao84 estimator for the number of species and its confidence limit, and for estimating the confidence limits for EH we estimated its standard error (SE) using the jackknife and derived confidence limits as 1.96 × SE. We used the NEBA data set for this demonstration; but we caution that that although the data are of the right form for the calculation they represent capture records of individual ants, not colonies as we have argued above would be more appropriate. Regression analyses were made using Statview 4.5 (Abacus Concepts).

## Results

**Figure 2:**
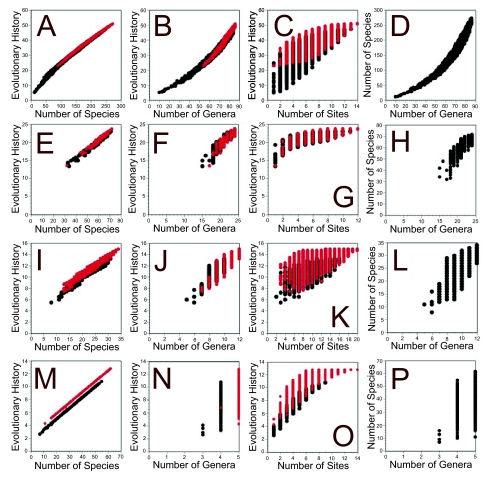
Relationships between species richness, generic richness, number of sites and evolutionary history preserved, and between the number of genera and number of species preserved, for the data sets of the flightless insects of North Queensland (A–D), Florida ants (E–H), New England bog ants (I–L) and New Zealand lepidosaurs (M–P). Where applicable, combinations of sites preserving a selected phylogenetically divergent taxon are given in red and others in black; the rare taxa are *Austrovelia queenslandica* (A–C), *Myrmecina americana* (E–G), *Amblyopone pallipes* (I–K) and the genus *Sphenodon* (M–O).

Graphs of species number and preserved evolutionary history (figure 2) show a strong relationship between these quantities. In every case there is a strong tendency for site combinations with the divergent taxa selected to preserve more evolutionary history than combinations with the same number of species but lacking these divergent taxa.

The number of genera is predictive of evolutionary history preserved (figure 2) but with the effect most marked when the number of genera is large (as in the NQFI data set). The relationship between evolutionary history preserved and the number of sites is often not a close one, but there is an evident significant payoff to selecting sites with the selected divergent taxa (figure 2). The advantage to selecting sites with these divergent taxa is marked for all data sets except FLA.

The relationship between number of species and number of genera varies between data sets, apparently in proportion to the range of numbers of genera preserved by different site combinations (figure 2). There is a very strong relationship for NQFI (a range of 10 to 86 genera preserved) and the weakest relationship is seen for the NZL data (three to five genera preserved).

Statistical analyses are problematic because each site enters into many site combinations, but regression analyses can be at least indicative. For each data set all three independent variables (number of sites, number of genera, number of species) were highly significant under multiple regression (Table 2) and all were retained in the model under stepwise regression (Table 3). For the stepwise regression, the order of entry of terms into the model was number of species > number of genera > number of sites for all data sets except NZL (with a very small number of genera), in which the order was number of species > number of sites > number of genera.

Because giving all results for the confidence limits for EH and species richness for all sites of an would make for a voluminous table, we present the results of all combinations of dropping one site at a time for the NEBA data, in Table 4.

## Discussion

We have demonstrated a method of using phylogenetic information implicit in systematic nomenclature to assess the conservation worth of sets of reserves using large proportions of their species, in fact potentially all of them. The method is not divorced from direct phylogenetic knowledge because systematists generally seek to make systematic nomenclature reflect this knowledge, and as it advances will modify the nomenclature. The information already being collected from surveys can be readily entered into the programs TREEMAKER and MeSA, and the results for moderate numbers of reserved (as in the NQFI case) readily sorted using popular spreadsheet programs such as EXCEL, enabling the most bio-diverse sets to be easily identified. The number of possible combinations does rise steeply with increasing number of locations, so that obtaining and listing all of these becomes prohibitive in computer time and effort, whether for identifying just species richness or EH. Simulated annealing has been proposed for identifying sets of locations maximising species richness (McDonnell et al. 2002) and this approach can also be used for maximising EH (Agapow & Crozier 2005).

The estimates of statistical sufficiency in Table 4 are not strictly correct for these data, as discussed above, but the results bring out an important point. For some sites 34 species were recorded and others 33, but the 22 combinations formed by dropping one site each time yielded results which did not differ significantly: all the various combinations are not significantly different with respect either to the number of species preserved or the EH. The management implication is that the criteria for choosing between those combinations which do not differ significantly can rest on other grounds than species richness or EH.

The identification of species is commonly a laborious and difficult process, so that it is natural that short cuts have been sought that avoid this task. One such short cut is “higher taxon richness”, in which higher taxa (such as genera or even families) are counted rather than species. Because higher taxa are more easily identified than species, this method is naturally attractive (reviewed by Crozier (1997)). In a study of subterranean bacterial communities related through an rRNA phylogeny, Crozier et al. (1999) found that higher taxon richness correlated well with GD. The present results indicate that the number of genera is highly predictive of EH (as gauged using systematic nomenclature) for large data sets. For small to medium sized data sets the predictiveness of EH drops off markedly as the range of number of genera preserved by site combinations decreases. For large data sets, such as NQFI, genus number is highly predictive of species number, a result suggesting that for such studies there could be a saving of effort through identifying specimens to genus only.

Phylogenies or surrogates based on systematic nomenclature have been used in or recommended for ecological studies on community structure (Warwick & Clarke 1994, 1998, Clarke & Warwick 2001, Webb et al. 2002, Cattin et al. 2004, Gotelli 2004), and estimated functional divergence has been used instead of phylogeny in examining community structure (Petchey & Gaston 2002, Petchey et al. 2004). There seems therefore to be a widespread move towards going beyond species richness in biodiversity assessment and similar endeavors, as also shown by the use of unit-length morphological phylogenies (Faith et al. 2004).

The methods suggested here have limitations. Groups in which there is minimal systematic structure, perhaps because they have radiated recently and not yet evolved high degrees of divergence, will have a poor reflection of phylogeny in their nomenclature. There are grounds for optimism, in that a study of the effects of phylogenetic inaccuracy on comparative analysis (Symonds 2002) found that the process is fairly robust against such errors. More serious, given the ambition to cover a significant proportion of the species in habitats (Humphries et al. 1995), is the lack of consistency across broad taxonomic groups, such as insects and mammals. If a consistent standard could be applied for systematics across at least the metazoa, such as a correspondence between systematic rank and time since origin (Avise & Johns 1999), then a broad array of animal groups could be included in such analyses. However, as it is, use of the NQFI data set shows that most terrestrial species could be included in analyses.

The argument in favor of a phylogenetic basis for setting conservation priorities was put persuasively by Wilson (1992) and implemented in various metrics by others (reviewed by Crozier (1997)). However the idea that the object of conservation is to preserve the widest diversity of features in the biota shows that a phylogenetic rationale has long been implicit. But even if the underlying rationale for biodiversity preservation is phylogenetic, need the methods for achieving it be? If large numbers of species are involved, does a phylogenetic approach to assessment still matter (Humphries et al. 1995, Crozier 1997)? Our results indicate that phylogeny (gauged through its surrogate of systematic nomenclature) will make the most difference when the number of species is small. However, given that it is much more difficult and labor-intensive to collect the data than to analyse them, it would seem negligent not to investigate the effects of phylogeny now that there are adequate tools for doing so.

## Figures and Tables

**Table 1:** Systematic nomenclature for some social bees (Michener 2000).

Subfamily	Tribe	Genus	Species
Apinae	Apini	*Apis*	*mellifera*
			*dorsata*
	Meliponini	*Melipona*	*beecheii*
		*Trigona*	*hypogea*

**Table 2:** ANOVA table for the four data sets, for the independent variables shown and the dependent variable Evolutionary History. The data sets are North Queensland Flightless Insects (NQFI), Florida Ants (FLA), New England Bog Ants (NEBA), and New Zealand Lepidosaurs (NZL). In each case the regression was significant with P < 0.0001. The regression in each case had 3 degrees of freedom and the total number of degrees of freedom is given after each dataset abbreviation.

Data/parameter	Coefficient	Standard Error	Standard Coefficient	t-value	P
**NQFI (16382)**					
Intercept	3.097	0.032	3.097	95.657	<0.0001
Number of Sites	−0.026	0.002	−0.008	−16.876	<0.0001
Number of Genera	0.163	0.001	0.209	197.165	<0.0001
Number of Species	0.125	<0.001	0.802	689.038	<0.0001
**FLA (4094)**					
Intercept	2.600	0.029	2.600	90.273	<0.0001
Number of Sites	0.011	0.001	0.014	8.241	<0.0001
Number of Genera	0.310	0.002	0.319	135.947	<0.0001
Number of Species	0.190	0.001	0.698	251.929	<0.0001
**NEBA (17464)**					
Intercept	2.417	0.021	2.427	115.492	<0.0001
Number of Sites	0.005	0.001	0.011	5.806	<0.0001
Number of Genera	0.333	0.003	0.272	119.329	<0.0001
Number of Species	0.252	0.001	0.758	296.172	<0.0001
**NZL (19729)**					
Intercept	−7.551	1.779	−7.551	−4.243	<0.0001
Number of Sites	0.115	0.007	0.185	16.224	<0.0001
Number of Genera	2.931	0.357	0.049	8.216	<0.0001
Number of Species	0.066	0.002	0.366	32.096	<0.0001

**Table 3:** Final ANOVA tables after all three independent variables (Number of Sites, Number of Genera and Number of Species) were entered into the stepwise regression analysis. In all cases the regressions were significant with P < 0.0001. The degrees of freedom were as given in Table 2. The adjusted R^2^ value for each regression is given in parentheses after each dataset abbreviation.

Data/parameter	Coefficient	Standard Error	Std. Coefficient	F-to-Remove
**NQFI (0.999)**					
Intercept	3.097	0.032	3.097	9150.323
Number of Sites	−0.026	0.002	−0.008	284.800
Number of Genera	0.163	0.001	0.209	38874.218
Number of Species	0.125	<0.001	0.802	474773.447
**FLA (0.994)**					
Intercept	2.600	0.029	2.600	8149.174
Number of Sites	0.011	0.001	0.014	67.909
Number of Genera	0.310	0.002	0.319	18481.488
Number of Species	0.190	0.001	0.698	63468.304
**NEBA (0.956)**					
Intercept	2.427	0.021	2.427	13338.354
Number of Sites	0.005	0.001	0.011	33.710
Number of Genera	0.333	0.003	0.272	14239.333
Number of Species	0.252	0.001	0.758	87717.638
**NZL (0.288)**					
Intercept	−7.551	1.779	−7.551	18.007
Number of Sites	0.115	0.007	0.185	263.220
Number of Genera	2.931	0.357	0.049	67.494
Number of Species	0.006	0.002	0.366	1030.168

**Table 4:** Confidence intervals for species richness calculated using the Chao84 estimator and for EH using the jackknife. The 22 combinations obtained by dropping each site in turn from Goltelli’s NEBA data are shown. For the data, see the Appendix.

Site Omitted	n	S	EH
ARC	34	37.961<46.000<70.349	13.419<15.000<16.581
BH	34	41.538<54.000<87.063	12.865<15.000<17.135
CB	33	34.597<39.000<55.548	13.250<14.750<16.250
CKB	33	46.566<57.000<75.458	13.177<14.500<15.823
HAW	34	37.962<46.000<70.349	13.419<15.000<16.581
HBC	33	36.962<45.000<69.349	13.169<14.750<16.331
OB	34	37.962<46.000<70.349	13.419<15.000<16.581
PK	34	37.961<46.000<70.349	13.419<15.000<16.581
QP	34	37.962<46.000<70.349	13.419<15.000<16.581
RP	33	40.538<53.000<86.063	13.092<14.750<16.408
SKP	33	34.597<39.000<55.548	13.276<14.500<15.724
SW	34	35.991<44.000<84.238	13.342<15.000<16.658
TPB	34	37.962<46.000<70.349	13.419<15.000<16.581
WIN	34	37.962<46.000<70.349	13.419<15.000<16.581
SPR	34	37.962<46.000<70.349	13.419<15.000<16.581
SNA	34	37.962<46.000<70.349	13.419<15.000<16.581
PEA	34	37.962<46.000<70.349	13.419<15.000<16.581
CHI	34	37.962<46.000<70.349	13.419<15.000<16.581
MOL	34	37.962<46.000<70.349	13.419<15.000<16.581
COL	33	36.962<45.000<69.349	13.169<14.750<16.331
CAR	34	37.962<46.000<70.349	13.419<15.000<16.581

**Terms:** n is the observed species richness, S the estimated value and its 95% confidence limits and EH is Evolutionary History and its 95% confidence limits.

## Appendix

NEXUS files for the analyses used in this paper. The trees involved can be readily viewed using TREE-VIEW X if the DATA block is removed or commented out.

#NEXUS

[! Created by TreeMakerB v1.0.8d 9/7/04]

[Data from Yeates, Bouchard & Monteith, 2002, who also give]

[the complete names for each species used]

[The systematic levels used were superorder, order, suborder, infraorder, superfamily, family, genus, and species]

BEGIN DATA;

DIMENSIONS NTAX=273 NCHAR=14;

FORMAT DATATYPE=CONTINUOUS;

CHARSTATELABELS

**Table:**

1	site_FU,
2	site_TU,
3	site_WU,
4	site_CU,
5	site_BM,
6	site_LU,
7	site_AU,
8	site_BK,
9	site_MT,
10	site_KU,
11	site_LE,
12	site_SU,
13	site_HU,
14	site_EU
;	

MATRIX

**Table:**

TargaremineA_LY08	1	1	0	0	0	0	0	0	0	0	0	0	0	0
Pseudignambia_D083	0	0	0	0	0	1	1	0	0	0	0	0	0	0
Myerslopella_ML06	0	0	0	0	0	0	0	1	0	0	0	0	0	0
Tryonicus_BL02	1	1	0	1	0	1	1	1	1	1	0	0	0	0
Notuchus_DE02	1	1	0	1	1	1	1	1	1	1	1	0	0	0
PeloridiidA_PE02	1	0	0	0	0	0	0	0	0	0	0	0	0	0
Hackeriella_PE01	0	0	0	0	0	0	0	1	0	0	0	0	0	0
Schizopteromiris_MI01	1	1	0	0	0	0	1	1	1	0	0	0	0	0
Grosshygia_GR03	0	0	0	1	0	0	0	0	0	0	0	0	0	0
Mesophloeobia_A078	0	1	0	0	0	0	0	0	0	0	0	0	0	0
Austrovelia_AV01	0	0	0	1	0	0	0	0	0	0	0	0	0	0
Kumaressa_A001	0	0	0	1	0	0	0	1	0	0	0	0	0	0
Aellocoris_A026	1	1	1	1	1	1	0	1	1	1	0	0	0	0
Euricoris_A027	0	0	1	1	1	1	1	1	1	1	1	1	1	0
Glyptoaptera_A030	0	0	0	0	0	0	0	0	0	0	0	0	0	1
Drakiessa_A066	0	0	0	0	0	0	0	0	0	0	0	0	0	1
GenusE_A041	1	0	0	1	0	0	0	0	0	0	0	0	0	0
GenusH_A043	0	0	0	0	0	1	1	0	0	0	0	0	0	0
Chelonoderus_A070	0	0	0	0	0	0	0	0	0	1	1	0	0	0
Aegisocoris_A072	0	1	0	1	0	0	0	0	0	0	0	0	0	0
Drakiessa_A088	0	0	0	1	0	0	0	0	0	0	0	0	0	0
Grosshygioides_GR04	1	0	0	0	0	0	0	0	0	0	0	0	0	0
Tomocoris_LY01	0	0	0	0	0	1	1	0	0	0	0	0	0	0
Australotarma_LY03	0	1	0	1	0	0	0	0	0	0	0	0	0	0
Targarops_LY06	0	1	0	1	0	0	0	0	0	0	0	0	0	0
Philipis_CP37	0	0	0	0	0	0	0	0	0	0	0	0	0	1
Darodilia_C069	0	0	0	0	0	0	1	0	0	0	0	0	0	0
TargaremineC_LY10	0	0	0	0	0	0	1	1	1	0	0	0	0	0
Mystropomus_C001	0	0	0	1	0	1	1	1	0	1	0	1	1	0
Pamborus_C005	0	0	0	0	0	0	1	1	0	0	0	0	0	0
Migadopine_C006	0	0	1	1	1	1	1	1	1	0	0	0	0	0
Laccopterum_C007	0	0	0	0	0	0	1	0	0	1	0	0	0	0
Mecyclothorax_C010	0	0	0	0	0	0	1	0	0	0	0	0	0	0
Raphetis_C011	0	0	0	1	0	0	1	1	0	0	0	0	0	0
Sitaphe_C012	0	1	1	1	0	1	1	1	1	1	0	0	0	0
Coptocarpus_C016	0	0	1	1	0	0	1	0	0	0	0	0	0	0
Illaphanus_C017	1	0	1	1	0	1	1	0	0	0	0	0	0	0
Castelnaudia_C021	0	0	0	0	0	0	0	0	0	0	0	1	1	0
Feronista_C022	0	0	0	1	0	0	0	0	0	0	0	0	0	0
Leiradira_C031	0	0	0	0	0	1	0	0	0	0	0	0	0	0
Loxogenius_C032	1	1	0	1	1	0	1	0	0	0	0	0	0	0
Notonomus_C043	0	0	0	0	0	0	1	0	0	0	0	0	0	0
Nurus_C044	0	0	0	0	0	0	0	0	0	0	0	0	0	1
Setalis_C046	0	0	0	0	0	1	1	1	0	0	0	0	0	0
Lecanomerus_C057	0	0	0	0	0	1	1	1	0	0	0	0	0	0
Harpaline_C060	0	0	0	0	0	0	0	0	0	0	0	0	0	1
Anomotarus_C063	1	1	0	0	0	0	0	0	0	0	0	0	0	0
Loxandrus_C065	0	0	0	0	0	0	0	0	0	0	1	0	1	0
Lacordairia_C067	0	0	0	0	0	1	1	1	0	0	0	0	0	0
Chariotheca_CM53	0	0	0	0	0	0	0	1	0	0	0	0	0	0
Terradessus_DY02	0	1	0	0	0	0	0	0	0	0	0	0	0	0
Athemistus_AT03	0	0	1	1	0	1	1	1	0	1	0	0	0	1
Blepegenes_AD01	0	0	0	1	0	1	1	1	0	1	0	0	0	0
Cardiothorax_AD02	0	0	1	1	0	1	0	0	0	0	0	0	0	0
Bluops_AD03	0	0	0	1	0	0	1	1	0	0	0	0	0	0
Adelium_AD14	0	0	0	0	0	0	1	0	0	0	0	0	0	0
Seirotrana_AD15	0	0	1	1	0	0	1	0	0	1	1	0	0	0
Adelium_AD18	0	0	0	0	0	0	0	1	1	0	0	0	0	0
Adelodemus_AD19	0	0	0	0	0	0	0	1	0	0	0	0	0	0
Bellendenum_AD22	0	0	0	0	0	0	0	1	1	0	0	0	0	0
Monteithium_AD24	0	0	0	1	0	0	0	0	0	0	0	0	0	0
Nolicima_AD25	0	0	0	1	0	0	1	1	0	1	0	0	0	0
Licinoma_AD26	0	0	0	0	0	0	0	0	0	0	0	1	0	0
Dicyrtodes_AD29	0	0	0	0	0	0	0	0	1	0	0	0	0	0
Epomidus_AD33	0	0	0	0	0	0	0	1	0	0	0	0	0	0
Diaspirus_AD34	0	0	0	0	0	0	1	0	0	0	0	0	0	0
Coripera_AD42	0	0	1	1	0	0	0	0	0	0	0	0	0	0
Dicyrtodes_AD49	0	0	0	0	0	1	0	0	0	0	0	0	0	0
Caxtonana_CM08	0	1	0	1	0	0	1	1	1	0	0	0	0	0
Apterotheca_CM11	0	0	0	1	0	0	0	0	0	0	0	0	0	0
Apterotheca_CM16	1	0	0	0	0	0	0	0	0	0	0	0	0	0
Hydissus_CM17	0	0	1	1	0	1	0	0	0	0	0	0	0	0
Apterotheca_CM51	0	0	0	0	0	0	0	0	0	1	0	0	0	0
Mychestes_MY05	1	1	1	1	1	1	1	1	0	1	0	0	0	0
Lissapterus_LU02	0	0	0	0	0	0	0	0	0	0	0	0	0	1
Amphistomus_D005	0	0	0	1	0	0	0	0	0	0	0	0	0	0
Aulacopris_D007	0	1	0	0	0	0	0	0	0	0	0	0	0	0
Aptenocanthon_D012	0	0	0	0	0	0	0	0	0	0	0	0	0	1
Temnoplectron_D065	0	0	0	0	0	0	0	0	0	0	0	1	1	0
Tryonicus_BL01	0	1	0	1	0	1	0	1	1	0	0	0	0	0
Myerslopella_ML01	0	0	0	1	0	1	0	0	0	0	0	0	0	0
Myerslopella_ML02	0	1	0	0	0	0	0	0	0	0	0	0	0	0
Myerslopella_ML03	1	0	0	0	0	0	0	0	0	0	0	0	0	0
Myerslopella_ML04	0	0	0	0	0	0	1	1	0	0	0	0	0	0
Myerslopella_ML05	0	0	0	1	0	0	0	0	0	0	0	0	0	0
Notuchus_DE01	0	0	0	0	0	0	0	1	0	0	0	0	0	0
Aellocoris_A019	0	0	0	0	0	1	0	1	0	0	0	0	0	0
Aellocoris_A020	0	0	0	1	0	0	0	0	0	0	0	0	0	0
Aellocoris_A021	0	0	0	0	0	0	1	0	0	1	0	1	0	1
Aellocoris_A022	1	1	0	1	0	0	0	0	0	0	0	0	0	0
Aellocoris_A023	1	0	0	0	0	0	0	0	0	0	0	0	0	0
Aellocoris_A024	0	1	0	0	0	0	0	0	0	0	0	0	0	0
Aellocoris_A025	1	1	1	1	0	0	0	0	0	0	0	0	0	0
Glyptoaptera_A028	0	0	0	1	1	1	1	1	0	0	0	0	0	0
Glyptoaptera_A029	0	1	0	1	0	0	1	1	1	1	0	0	0	0
Spinandra_A031	1	1	0	0	1	1	1	1	1	0	1	0	0	0
Spinandra_A032	0	0	0	0	0	0	0	1	0	0	0	0	0	0
Spinandra_A033	0	0	0	0	0	0	0	1	1	0	0	0	0	0
Spinandra_A034	0	0	0	0	0	0	1	0	0	1	0	0	0	0
GenusA_A037	1	1	1	1	1	1	1	1	1	0	0	0	0	0
GenusB_A038	0	0	0	0	0	0	1	1	0	1	0	0	0	0
GenusA_A035	0	1	1	1	0	0	0	0	0	0	0	0	0	0
GenusA_A036	0	0	0	0	0	0	1	1	0	0	0	0	0	0
GenusE_A039	0	1	0	1	1	1	1	0	0	1	0	0	1	0
GenusE_A040	0	0	0	1	0	0	0	0	0	0	0	0	0	0
Drakiessa_A064	0	0	0	0	0	0	1	1	0	0	0	0	0	0
Drakiessa_A065	0	0	0	0	0	1	0	1	1	0	0	0	0	0
Chelonoderus_A067	1	1	1	1	1	1	0	1	1	0	0	0	0	0
Chelonoderus_A068	0	0	0	0	0	0	1	0	0	0	0	0	0	0
Chelonoderus_A069	0	0	0	0	0	0	1	1	0	0	0	0	0	0
Aegisocoris_A071	0	0	0	0	0	0	1	1	1	0	0	0	0	0
Neophloeobia_A073	0	0	0	0	0	0	0	0	0	0	0	1	1	0
Neophloeobia_A074	1	1	0	1	1	1	0	1	0	0	0	0	0	0
Neophloeobia_A075	0	0	0	0	0	0	0	0	0	0	1	0	0	0
Mesophloeobia_A077	0	0	0	0	0	0	0	0	0	1	0	0	0	0
Granulaptera_A079	0	0	1	1	1	1	1	1	1	0	0	0	0	0
Granulaptera_A080	0	1	0	0	1	0	1	1	1	1	0	0	0	0
Granulaptera_A081	0	0	0	0	0	0	1	1	1	1	0	0	0	0
Granulaptera_A082	0	1	0	1	0	0	1	0	0	0	0	0	0	0
Granulaptera_A083	0	0	0	0	0	1	1	1	0	0	0	0	0	0
Granulaptera_A084	1	1	0	0	0	0	0	0	0	0	0	0	0	0
Grosshygia_GR01	0	0	0	0	0	0	1	0	0	0	0	0	0	0
Grosshygia_GR02	0	0	0	0	0	0	0	1	1	0	0	0	0	0
TargaremineA_LY07	0	0	0	1	0	0	1	1	1	0	0	0	0	0
Pamborus_C003	1	1	1	1	1	1	1	1	0	1	1	1	1	0
Pamborus_C004	0	0	1	1	0	1	0	0	0	0	0	0	0	0
Mecyclothorax_C008	0	0	0	0	0	1	0	1	0	0	0	0	0	0
Mecyclothorax_C009	0	1	0	1	0	0	0	0	0	0	0	0	0	0
Coptocarpus_C015	0	0	0	0	0	0	1	1	0	0	0	0	0	0
Castelnaudia_C018	0	0	0	0	0	0	0	0	0	1	0	0	0	0
Castelnaudia_C019	1	1	1	1	1	0	0	0	0	0	0	0	0	0
Castelnaudia_C020	0	0	0	0	0	1	1	1	0	1	0	0	0	0
Leiradira_C023	0	0	0	0	0	0	1	1	0	0	0	0	0	0
Leiradira_C024	0	0	0	0	0	0	0	1	0	0	0	0	0	0
Leiradira_C025	0	0	0	0	0	1	0	0	0	0	0	0	0	0
Leiradira_C026	1	1	0	1	1	1	0	0	0	0	0	0	0	0
Leiradira_C027	0	0	0	0	0	0	1	1	1	0	0	0	0	0
Leiradira_C028	1	0	0	0	0	0	0	0	0	0	0	0	0	0
Leiradira_C029	0	1	0	1	0	0	0	0	0	0	0	0	0	0
Leiradira_C030	0	0	0	1	0	0	0	0	0	0	0	0	0	0
Notonomus_C033	0	0	0	1	0	0	0	0	0	0	0	0	0	0
Notonomus_C034	0	0	0	1	0	0	1	0	0	1	1	1	1	0
Notonomus_C035	0	0	0	0	0	0	0	0	0	0	0	0	0	1
Notonomus_C036	0	0	0	1	0	0	0	0	0	0	0	0	0	0
Notonomus_C037	0	0	0	0	0	0	1	1	0	0	0	0	0	0
Notonomus_C038	0	0	0	0	0	0	0	1	0	0	0	0	0	0
Notonomus_C039	0	0	0	0	0	1	1	1	0	0	0	0	0	0
Notonomus_C040	0	0	0	0	0	1	1	0	0	0	0	0	0	0
Notonomus_C041	0	1	1	1	0	0	0	0	0	0	0	0	0	0
Notonomus_C042	0	0	1	0	0	0	0	0	0	0	0	0	0	0
Trichosternus_C047	0	0	0	0	0	0	1	0	0	0	0	0	0	0
Trichosternus_C048	0	0	0	0	0	0	0	1	0	0	0	0	0	0
Trichosternus_C049	0	0	0	1	1	1	1	0	0	1	0	0	0	0
Trichosternus_C050	0	0	0	0	0	0	0	1	0	0	0	0	0	0
Trichosternus_C051	0	0	0	0	0	0	1	0	0	0	0	0	0	0
Trichosternus_C052	0	0	0	0	0	0	0	0	0	0	0	1	1	0
Trichosternus_C053	0	0	0	0	0	0	1	1	0	0	0	0	0	0
Trichosternus_C055	0	0	0	0	0	0	0	0	0	0	0	0	0	1
Trichosternus_C056	0	0	0	0	0	1	0	0	0	0	0	0	0	0
Harpaline_C058	1	0	0	0	0	0	0	0	0	0	0	0	0	0
Harpaline_C059	0	1	0	1	0	0	0	0	0	0	0	0	0	0
Anomotarus_C061	0	0	0	1	0	0	0	0	0	1	0	0	0	0
Carenum_C068	0	0	1	0	0	1	1	1	0	0	0	0	0	0
Philipis_CP07	0	0	0	0	0	0	0	1	1	0	0	0	0	0
Philipis_CP10	1	0	0	0	0	0	0	0	0	0	0	0	0	0
Philipis_CP15	0	1	0	0	0	0	0	0	0	0	0	0	0	0
Philipis_CP16	0	1	1	0	0	0	0	0	0	0	0	0	0	0
Philipis_CP19	0	1	0	0	0	0	0	0	0	0	0	0	0	0
Philipis_CP20	0	0	0	1	0	0	0	0	0	0	0	0	0	0
Philipis_CP21	0	0	0	0	0	0	0	1	0	0	0	0	0	0
Philipis_CP23	0	0	0	0	0	0	0	1	0	0	0	0	0	0
Philipis_CP24	0	0	0	0	0	0	0	1	0	0	0	0	0	0
Philipis_CP25	0	0	0	0	0	0	0	1	0	0	0	0	0	0
Philipis_CP26	0	0	0	0	0	0	0	0	0	0	0	0	0	1
Philipis_CP27	0	0	0	0	0	1	0	0	0	0	0	0	0	0
Philipis_CP32	0	0	0	0	0	0	1	1	0	0	0	0	0	0
Philipis_CP33	0	0	0	1	0	0	0	0	0	0	0	0	0	0
Philipis_CP34	0	0	0	0	0	1	0	1	1	0	0	0	0	0
Philipis_CP36	0	0	0	0	0	0	0	0	1	0	0	0	0	0
Terradessus_DY01	0	1	0	0	0	0	0	0	0	0	0	0	0	0
Athemistus_AT01	0	0	0	1	0	0	0	0	0	0	0	0	0	0
Athemistus_AT02	1	0	0	0	0	0	0	0	0	0	0	0	0	0
Adelium_AD04	1	0	0	1	1	1	1	1	0	1	1	0	0	0
Adelium_AD06	0	1	1	1	0	1	1	1	0	0	0	0	0	0
Adelium_AD07	1	1	0	1	1	0	0	0	0	0	0	0	0	0
Adelium_AD08	0	1	0	1	1	1	1	1	1	1	1	0	0	0
Adelium_AD09	0	0	0	0	0	0	0	0	0	0	1	1	1	1
Adelium_AD11	0	0	0	0	0	0	0	0	0	0	0	0	0	1
Adelium_AD12	0	0	0	0	0	0	0	0	0	0	0	1	1	0
Adelium_AD13	0	0	0	0	0	0	1	0	0	0	0	0	0	0
Coripera_AD16	1	1	0	0	0	0	0	0	0	0	0	0	0	0
Adelium_AD17	1	0	0	1	0	1	1	1	0	1	0	0	0	0
Bellendenum_AD20	0	0	0	0	0	0	1	0	0	0	0	0	0	0
Bellendenum_AD21	0	0	0	0	0	0	0	1	0	0	0	0	0	0
Monteithium_AD23	1	1	0	0	0	0	0	0	0	0	0	0	0	0
Dicyrtodes_AD28	0	0	0	0	0	0	0	1	0	0	0	0	0	0
Epomidus_AD30	0	0	0	0	0	0	0	1	0	0	0	0	0	0
Diaspirus_AD31	0	0	0	0	0	0	1	0	0	0	0	0	0	0
Leptogastrus_AD35	0	0	0	1	0	0	0	0	0	0	0	0	0	0
Leptogastrus_AD36	1	1	0	0	0	0	0	0	0	0	0	0	0	0
Coripera_AD40	0	0	0	0	0	1	0	0	0	0	0	0	0	0
Coripera_AD41	0	0	0	1	0	0	0	0	0	0	0	0	0	0
Apocryphodes_AD43	0	0	0	0	0	0	0	0	1	0	0	0	0	0
Leptogastrus_AD44	0	0	1	1	0	0	0	0	0	0	0	0	0	0
Adelium_AD45	0	0	0	0	0	0	1	0	0	0	0	0	0	0
Bellendenum_AD47	1	1	0	0	0	0	0	0	0	0	0	0	0	0
Caxtonana_CM01	0	0	0	0	0	0	1	0	0	0	0	0	0	0
Caxtonana_CM02	0	0	0	1	0	0	0	0	0	0	0	0	0	0
Caxtonana_CM03	0	0	0	0	0	0	1	0	0	0	0	0	0	0
Caxtonana_CM04	0	0	0	0	0	0	0	1	0	0	0	0	0	0
Caxtonana_CM05	0	0	0	0	0	0	0	0	1	0	0	0	0	0
Caxtonana_CM06	0	0	0	0	0	0	1	1	0	0	0	0	0	0
Caxtonana_CM07	0	0	1	1	0	0	0	0	0	0	0	0	0	0
Apterotheca_CM09	0	0	0	0	0	0	0	1	0	0	0	0	0	0
Apterotheca_CM10	0	0	0	1	0	0	0	0	0	0	0	0	0	0
Cuemus_CM12	1	0	0	0	0	0	0	0	0	0	0	0	0	0
Cuemus_CM13	0	0	0	0	0	0	0	1	1	0	0	0	0	0
Caxtonana_CM14	1	0	0	0	0	0	0	0	0	0	0	0	0	0
Caxtonana_CM15	0	0	0	0	0	0	1	0	0	0	0	0	0	0
Omolipus_CM18	1	1	1	1	1	1	0	1	0	0	0	0	0	0
Caxtonana_CM21	0	0	0	0	0	0	0	0	0	1	0	0	0	0
Apterotheca_CM23	0	0	0	1	0	1	1	1	0	0	0	0	0	0
Apterotheca_CM24	0	0	0	0	0	0	1	1	0	0	0	0	0	0
Apterotheca_CM25	0	1	1	0	1	1	0	0	0	1	0	0	0	0
Apterotheca_CM26	0	0	0	1	0	0	0	0	0	0	0	0	0	0
Apterotheca_CM27	0	0	0	0	0	0	1	1	0	0	0	0	0	0
Apterotheca_CM28	0	0	0	0	0	0	0	0	0	0	0	1	1	0
Apterotheca_CM29	0	0	0	0	0	0	0	0	0	0	0	0	0	1
Apterotheca_CM30	0	1	0	0	0	0	0	0	0	0	0	0	0	0
Apterotheca_CM32	0	0	0	0	0	0	1	0	0	0	0	0	0	0
Apterotheca_CM33	0	0	0	0	0	1	0	0	0	0	0	0	0	0
Apterotheca_CM35a	0	0	0	0	0	0	0	0	1	0	0	0	0	0
Apterotheca_CM35b	0	0	0	0	1	0	0	0	0	0	0	0	0	0
Apterotheca_CM35c	1	0	0	0	0	0	0	0	0	0	0	0	0	0
Apterotheca_CM36	0	0	0	0	0	0	0	1	0	0	0	0	0	0
Apterotheca_CM37	0	0	0	0	1	0	0	0	0	0	0	0	0	0
Apterotheca_CM38	0	0	0	0	0	0	1	0	0	1	0	0	0	0
Apterotheca_CM39	0	1	0	1	0	0	0	0	0	0	0	0	0	0
Apterotheca_CM40	1	0	0	0	0	0	0	0	0	0	0	0	0	0
Caxtonana_CM41	0	0	0	0	0	0	0	0	1	0	0	0	0	0
Apterotheca_CM42	0	0	0	1	0	0	0	0	0	0	0	0	0	0
Omolipus_CM43	0	0	0	1	0	0	0	0	0	0	0	0	0	0
Apterotheca_CM50	0	0	0	0	0	1	1	0	0	0	0	0	0	0
Mychestes_MY01	1	1	0	1	0	1	1	1	1	0	0	0	0	0
Mychestes_MY03	0	0	0	0	0	0	0	1	0	0	0	0	0	0
Lissapterus_LU01	0	1	0	0	0	0	0	1	0	0	0	0	0	0
Amphistomus_D004	0	0	0	0	0	0	0	0	0	0	0	1	0	0
Aptenocanthon_D008	0	0	0	0	0	1	1	1	0	0	0	0	0	0
Aptenocanthon_D009	0	0	0	0	0	0	1	1	0	1	0	0	0	0
Aptenocanthon_D010	1	0	0	0	0	0	0	0	0	0	0	0	0	0
Aptenocanthon_D011	0	0	0	0	0	1	0	0	0	0	0	0	0	0
Temnoplectron_D019	0	0	0	1	1	0	0	0	0	0	0	0	0	0
Lepanus_D088	0	0	0	0	0	0	0	0	0	0	0	0	0	1
Temnoplectron_D097	1	0	0	0	0	0	0	0	0	0	0	0	0	0
Temnoplectron_D138	0	1	0	0	0	0	0	0	0	0	0	0	0	0
Pseudignambia_D139	0	0	0	1	0	0	0	0	0	0	0	0	0	0
Pseudignambia_D140	0	0	0	0	0	0	1	0	0	0	0	0	0	0
Pseudignambia_D141	0	0	0	0	0	0	0	1	0	0	0	0	0	0
Pseudignambia_D142	0	1	0	0	0	0	0	0	0	0	0	0	0	0
Pseudignambia_D143	0	0	0	0	0	0	0	1	0	0	0	0	0	0
Pseudignambia_D144	0	1	0	1	0	0	0	0	0	0	0	0	0	0
Pseudignambia_D145	0	0	0	0	0	0	0	1	0	0	0	0	0	0
Pseudignambia_D146	0	0	0	0	0	0	0	0	0	1	0	0	0	0
Pseudignambia_D147	0	0	1	1	0	0	0	0	0	0	0	0	0	0
Pseudignambia_D148	0	0	0	0	0	0	1	1	0	0	0	0	0	0
Pseudignambia_D149	0	0	0	0	1	1	0	0	0	0	0	0	0	0
Pseudignambia_D150	0	0	0	0	0	0	0	1	0	0	0	0	0	0
Pseudignambia_D151	0	0	0	0	0	1	1	0	0	0	0	0	0	0
Pseudignambia_D152	0	0	0	0	0	0	0	0	1	0	0	0	0	0
Pseudignambia_D153	0	0	0	0	0	0	0	0	1	0	0	0	0	0
Pseudignambia_D154	0	0	0	0	0	0	0	0	1	0	0	0	0	0
Pseudignambia_D155	0	0	0	0	0	0	0	0	1	0	0	0	0	0
Pseudignambia_D156	0	0	0	0	0	1	0	0	0	0	0	0	0	0
;														

END;

BEGIN TREES;

TRANSLATE

**Table:**

203	TargaremineA_LY08,
388	Pseudignambia_D083,
138	Myerslopella_ML06,
132	Tryonicus_BL02,
140	Notuchus_DE02,
143	PeloridiidA_PE02,
142	Hackeriella_PE01,
141	Schizopteromiris_MI01,
197	Grosshygia_GR03,
187	Mesophloeobia_A078,
144	Austrovelia_AV01,
145	Kumaressa_A001,
153	Aellocoris_A026,
154	Euricoris_A027,
157	Glyptoaptera_A030,
174	Drakiessa_A066,
171	GenusE_A041,
175	GenusH_A043,
179	Chelonoderus_A070,
181	Aegisocoris_A072,
194	Drakiessa_A088,
198	Grosshygioides_GR04,
199	Tomocoris_LY01,
200	Australotarma_LY03,
201	Targarops_LY06,
284	Philipis_CP37,
267	Darodilia_C069,
204	TargaremineC_LY10,
205	Mystropomus_C001,
208	Pamborus_C005,
209	Migadopine_C006,
210	Laccopterum_C007,
213	Mecyclothorax_C010,
214	Raphetis_C011,
215	Sitaphe_C012,
217	Coptocarpus_C016,
218	Illaphanus_C017,
222	Castelnaudia_C021,
223	Feronista_C022,
232	Leiradira_C031,
233	Loxogenius_C032,
244	Notonomus_C043,
245	Nurus_C044,
246	Setalis_C046,
257	Lecanomerus_C057,
260	Harpaline_C060,
262	Anomotarus_C063,
263	Loxandrus_C065,
264	Lacordairia_C067,
372	Chariotheca_CM53,
286	Terradessus_DY02,
289	Athemistus_AT03,
290	Blepegenes_AD01,
291	Cardiothorax_AD02,
292	Bluops_AD03,
301	Adelium_AD14,
302	Seirotrana_AD15,
305	Adelium_AD18,
306	Adelodemus_AD19,
309	Bellendenum_AD22,
311	Monteithium_AD24,
312	Nolicima_AD25,
313	Licinoma_AD26,
315	Dicyrtodes_AD29,
317	Epomidus_AD33,
319	Diaspirus_AD34,
324	Coripera_AD42,
329	Dicyrtodes_AD49,
337	Caxtonana_CM08,
340	Apterotheca_CM11,
345	Apterotheca_CM16,
346	Hydissus_CM17,
371	Apterotheca_CM51,
375	Mychestes_MY05,
377	Lissapterus_LU02,
379	Amphistomus_D005,
380	Aulacopris_D007,
385	Aptenocanthon_D012,
387	Temnoplectron_D065,
131	Tryonicus_BL01,
133	Myerslopella_ML01,
134	Myerslopella_ML02,
135	Myerslopella_ML03,
136	Myerslopella_ML04,
137	Myerslopella_ML05,
139	Notuchus_DE01,
146	Aellocoris_A019,
147	Aellocoris_A020,
148	Aellocoris_A021,
149	Aellocoris_A022,
150	Aellocoris_A023,
151	Aellocoris_A024,
152	Aellocoris_A025,
155	Glyptoaptera_A028,
156	Glyptoaptera_A029,
158	Spinandra_A031,
159	Spinandra_A032,
160	Spinandra_A033,
161	Spinandra_A034,
167	GenusA_A037,
168	GenusB_A038,
165	GenusA_A035,
166	GenusA_A036,
169	GenusE_A039,
170	GenusE_A040,
172	Drakiessa_A064,
173	Drakiessa_A065,
176	Chelonoderus_A067,
177	Chelonoderus_A068,
178	Chelonoderus_A069,
180	Aegisocoris_A071,
182	Neophloeobia_A073,
183	Neophloeobia_A074,
184	Neophloeobia_A075,
186	Mesophloeobia_A077,
188	Granulaptera_A079,
189	Granulaptera_A080,
190	Granulaptera_A081,
191	Granulaptera_A082,
192	Granulaptera_A083,
193	Granulaptera_A084,
195	Grosshygia_GR01,
196	Grosshygia_GR02,
202	TargaremineA_LY07,
206	Pamborus_C003,
207	Pamborus_C004,
211	Mecyclothorax_C008,
212	Mecyclothorax_C009,
216	Coptocarpus_C015,
219	Castelnaudia_C018,
220	Castelnaudia_C019,
221	Castelnaudia_C020,
224	Leiradira_C023,
225	Leiradira_C024,
226	Leiradira_C025,
227	Leiradira_C026,
228	Leiradira_C027,
229	Leiradira_C028,
230	Leiradira_C029,
231	Leiradira_C030,
234	Notonomus_C033,
235	Notonomus_C034,
236	Notonomus_C035,
237	Notonomus_C036,
238	Notonomus_C037,
239	Notonomus_C038,
240	Notonomus_C039,
241	Notonomus_C040,
242	Notonomus_C041,
243	Notonomus_C042,
247	Trichosternus_C047,
248	Trichosternus_C048,
249	Trichosternus_C049,
250	Trichosternus_C050,
251	Trichosternus_C051,
252	Trichosternus_C052,
253	Trichosternus_C053,
254	Trichosternus_C055,
255	Trichosternus_C056,
258	Harpaline_C058,
259	Harpaline_C059,
261	Anomotarus_C061,
265	Carenum_C068,
268	Philipis_CP07,
269	Philipis_CP10,
270	Philipis_CP15,
271	Philipis_CP16,
272	Philipis_CP19,
273	Philipis_CP20,
274	Philipis_CP21,
275	Philipis_CP23,
276	Philipis_CP24,
277	Philipis_CP25,
278	Philipis_CP26,
279	Philipis_CP27,
280	Philipis_CP32,
281	Philipis_CP33,
282	Philipis_CP34,
283	Philipis_CP36,
285	Terradessus_DY01,
287	Athemistus_AT01,
288	Athemistus_AT02,
293	Adelium_AD04,
294	Adelium_AD06,
295	Adelium_AD07,
296	Adelium_AD08,
297	Adelium_AD09,
298	Adelium_AD11,
299	Adelium_AD12,
300	Adelium_AD13,
303	Coripera_AD16,
304	Adelium_AD17,
307	Bellendenum_AD20,
308	Bellendenum_AD21,
310	Monteithium_AD23,
314	Dicyrtodes_AD28,
316	Epomidus_AD30,
318	Diaspirus_AD31,
320	Leptogastrus_AD35,
321	Leptogastrus_AD36,
322	Coripera_AD40,
323	Coripera_AD41,
325	Apocryphodes_AD43,
326	Leptogastrus_AD44,
327	Adelium_AD45,
328	Bellendenum_AD47,
330	Caxtonana_CM01,
331	Caxtonana_CM02,
332	Caxtonana_CM03,
333	Caxtonana_CM04,
334	Caxtonana_CM05,
335	Caxtonana_CM06,
336	Caxtonana_CM07,
338	Apterotheca_CM09,
339	Apterotheca_CM10,
341	Cuemus_CM12,
342	Cuemus_CM13,
343	Caxtonana_CM14,
344	Caxtonana_CM15,
347	Omolipus_CM18,
348	Caxtonana_CM21,
349	Apterotheca_CM23,
350	Apterotheca_CM24,
351	Apterotheca_CM25,
352	Apterotheca_CM26,
353	Apterotheca_CM27,
354	Apterotheca_CM28,
355	Apterotheca_CM29,
356	Apterotheca_CM30,
357	Apterotheca_CM32,
358	Apterotheca_CM33,
359	Apterotheca_CM35a,
360	Apterotheca_CM35b,
361	Apterotheca_CM35c,
362	Apterotheca_CM36,
363	Apterotheca_CM37,
364	Apterotheca_CM38,
365	Apterotheca_CM39,
366	Apterotheca_CM40,
367	Caxtonana_CM41,
368	Apterotheca_CM42,
369	Omolipus_CM43,
370	Apterotheca_CM50,
373	Mychestes_MY01,
374	Mychestes_MY03,
376	Lissapterus_LU01,
378	Amphistomus_D004,
381	Aptenocanthon_D008,
382	Aptenocanthon_D009,
383	Aptenocanthon_D010,
384	Aptenocanthon_D011,
386	Temnoplectron_D019,
389	Lepanus_D088,
390	Temnoplectron_D097,
391	Temnoplectron_D138,
392	Pseudignambia_D139,
393	Pseudignambia_D140,
394	Pseudignambia_D141,
395	Pseudignambia_D142,
396	Pseudignambia_D143,
397	Pseudignambia_D144,
398	Pseudignambia_D145,
399	Pseudignambia_D146,
400	Pseudignambia_D147,
401	Pseudignambia_D148,
402	Pseudignambia_D149,
403	Pseudignambia_D150,
404	Pseudignambia_D151,
405	Pseudignambia_D152,
406	Pseudignambia_D153,
407	Pseudignambia_D154,
408	Pseudignambia_D155,
409	Pseudignambia_D156
;	

TREE tree_1 = ((((((((131:0.125,132:0.125):0.125):0.125):0.125):0.125):0.125):0.125):0.125, (((((((133:0.125,134:0.125,135:0.125,136:0.125,137:0.125,138:0.125):0.125):0.125):0.125): 0.125,((((139:0.125,140:0.125):0.125):0.125):0.125):0.125):0.125, (((((141:0.125):0.125):0.125):0.125):0.125,((((142:0.125):0.125, (143:0.125):0.125):0.125):0.125):0.125,((((144:0.125):0.125):0.125):0.125):0.125, ((((145:0.125):0.125, (146:0.125,147:0.125,148:0.125,149:0.125,150:0.125,151:0.125,152:0.125,153:0.125):0.125, (154:0.125):0.125,(155:0.125,156:0.125,157:0.125):0.125, (158:0.125,159:0.125,160:0.125,161:0.125):0.125,(165:0.125,166:0.125,167:0.125):0.125, (172:0.125,173:0.125,174:0.125,194:0.125):0.125, (176:0.125,177:0.125,178:0.125,179:0.125):0.125,(180:0.125,181:0.125):0.125, (182:0.125,183:0.125,184:0.125):0.125, (188:0.125,189:0.125,190:0.125,191:0.125,192:0.125,193:0.125):0.125,(168:0.125):0.125, (169:0.125,170:0.125,171:0.125):0.125,(175:0.125):0.125, (186:0.125,187:0.125):0.125):0.125):0.125,(((195:0.125,196:0.125,197:0.125):0.125, (198:0.125):0.125):0.125):0.125,(((199:0.125):0.125,(200:0.125):0.125,(201:0.125):0.125, (202:0.125,203:0.125):0.125,(204:0.125):0.125):0.125):0.125):0.125):0.125):0.125):0.125, (((((((205:0.125):0.125,(206:0.125,207:0.125,208:0.125):0.125,(209:0.125):0.125, (210:0.125):0.125,(211:0.125,212:0.125,213:0.125):0.125,(214:0.125):0.125, (215:0.125):0.125,(216:0.125,217:0.125):0.125,(218:0.125):0.125, (219:0.125,220:0.125,221:0.125,222:0.125):0.125,(223:0.125):0.125, (224:0.125,225:0.125,226:0.125,227:0.125,228:0.125,229:0.125,230:0.125,231:0.125,232:0.125):0.125,(233:0.125):0.125, (234:0.125,235:0.125,236:0.125,237:0.125,238:0.125,239:0.125,240:0.125,241:0.125,242:0.125,243:0.125,244:0.125):0.125,(245:0.125):0.125,(246:0.125):0.125, (247:0.125,248:0.125,249:0.125,250:0.125,251:0.125,252:0.125,253:0.125,254:0.125,255:0.125):0.125,(258:0.125,259:0.125,260:0.125):0.125,(261:0.125,262:0.125):0.125, (263:0.125):0.125,(264:0.125):0.125,(265:0.125):0.125, (268:0.125,269:0.125,270:0.125,271:0.125,272:0.125,273:0.125,274:0.125,275:0.125,276:0.125,277:0.125,278:0.125,279:0.125,280:0.125,281:0.125,282:0.125,283:0.125,284:0.125):0.125, (257:0.125):0.125,(267:0.125):0.125):0.125, ((285:0.125,286:0.125):0.125):0.125):0.125):0.125):0.125, (((((287:0.125,288:0.125,289:0.125):0.125):0.125):0.125,(((290:0.125):0.125, (291:0.125):0.125,(292:0.125):0.125, (293:0.125,294:0.125,295:0.125,296:0.125,297:0.125,298:0.125,299:0.125,300:0.125,301:0.125,304:0.125,305:0.125,327:0.125):0.125,(302:0.125):0.125, (303:0.125,322:0.125,323:0.125,324:0.125):0.125,(306:0.125):0.125, (307:0.125,308:0.125,309:0.125,328:0.125):0.125,(310:0.125,311:0.125):0.125, (312:0.125):0.125,(313:0.125):0.125,(314:0.125,315:0.125,329:0.125):0.125, (316:0.125,317:0.125):0.125,(318:0.125,319:0.125):0.125, (320:0.125,321:0.125,326:0.125):0.125,(325:0.125):0.125, (330:0.125,331:0.125,332:0.125,333:0.125,334:0.125,335:0.125,336:0.125,337:0.125,343:0.125,344:0.125,348:0.125,367:0.125):0.125, (338:0.125,339:0.125,340:0.125,345:0.125,349:0.125,350:0.125,351:0.125,352:0.125,353:0.125,354:0.125,355:0.125,356:0.125,357:0.125,358:0.125,359:0.125,360:0.125,361:0.125,362:0.125,363:0.125,364:0.125,365:0.125,366:0.125,368:0.125,370:0.125,371:0.125):0.125, (341:0.125,342:0.125):0.125,(346:0.125):0.125,(347:0.125,369:0.125):0.125, (372:0.125):0.125,(373:0.125,374:0.125,375:0.125):0.125):0.125):0.125):0.125, ((((376:0.125,377:0.125):0.125):0.125,((378:0.125,379:0.125):0.125,(380:0.125):0.125, (381:0.125,382:0.125,383:0.125,384:0.125,385:0.125):0.125, (386:0.125,387:0.125,390:0.125,391:0.125):0.125, (388:0.125,392:0.125,393:0.125,394:0.125,395:0.125,396:0.125,397:0.125,398:0.125,399:0.125,400:0.125,401:0.125,402:0.125,403:0.125,404:0.125,405:0.125,406:0.125,407:0.125,408:0.125,409:0.125):0.125,(389:0.125):0.125):0.125):0.125):0.125):0.125):0.125):0.125); END;

#NEXUS

[! Created by TreeMakerB v1.0.7d 12/4/04]

[Data courtesy of NJ Gottelli from the study by Gottelli & Ellison 2002]

[Data are from ‘Most Recent Bog Ant Matrices.xls’]

[panel ‘Forests. Abundance data from pitfall traps only]

[The systematic divisions used were subfamily, tribe, genus and species]

BEGIN DATA;

DIMENSIONS NTAX=34 NCHAR=22;

FORMAT DATATYPE=CONTINUOUS;

CHARSTATELABELS

**Table:**

1	site_ARC,
2	site_BH,
3	site_CB,
4	site_CKB,
5	site_HAW,
6	site_HBC,
7	site_OB,
8	site_PK,
9	site_QP,
10	site_RP,
11	site_SKP,
12	site_SW,
13	site_TPB,
14	site_WIN,
15	site_SPR,
16	site_SNA,
17	site_PEA,
18	site_CHI,
19	site_MOL,
20	site_COL,
21	site_MOO,
22	site_CAR
;	

MATRIX

**Table:**

Amblypone_pallipes	0	0	0	0	0	1	0	0	0	0	3	0	0	0	0	0	0	0	6	0	0	0
Aphaenogaster_rudis	48	1	2	63	15	98	41	5	5	22	35	16	2	0	11	0	0	7	0	23	0	0
Leptothorax_curvispinosus	0	0	0	0	0	0	0	0	0	3	8	0	0	0	0	0	0	0	0	0	0	0
Leptothorax_ambiguus	0	0	0	0	0	0	0	0	0	0	0	0	0	0	0	0	0	0	0	4	0	0
Leptothorax_longispinosus	0	18	2	3	0	0	16	1	0	1	1	0	0	0	5	0	0	0	0	16	0	0
Myrmecina_americana	0	2	0	0	0	1	0	0	0	0	0	0	0	0	0	0	0	0	0	0	0	0
Myrmica_cfbrevispinosus	0	0	0	4	0	0	0	0	0	0	0	0	0	0	0	1	0	0	0	0	0	0
Myrmica_detrinodis	0	1	0	0	0	0	0	0	1	0	0	0	0	0	0	0	1	0	0	0	59	12
Myrmica_punctiventris	2	0	115	44	0	50	1	17	1	202	8	16	1	1	6	1	0	0	0	139	0	0
Myrmica_sculptilis	0	2	1	24	0	9	0	1	23	60	7	4	0	0	0	0	0	0	0	0	0	0
Myrmica_smithana	0	0	0	5	0	3	0	0	1	56	0	1	0	0	0	0	0	0	0	0	0	0
Stenamma_diecki	0	3	0	0	0	0	11	0	0	0	0	1	1	0	0	0	0	1	0	0	1	0
Stenamma_impar	0	1	0	4	0	0	0	0	0	0	0	1	0	0	3	0	0	0	0	2	0	0
Stenamma_schmitti	0	1	1	0	0	1	1	0	0	0	0	1	0	0	0	0	0	0	0	0	0	0
Camponotus_herculeanus	0	1	0	0	0	0	0	0	1	0	1	0	0	0	0	0	0	3	3	0	5	39
Camponotus_noveborencensis	0	0	0	0	0	1	1	1	4	0	0	0	0	4	0	1	0	0	0	0	0	0
Camponotus_nearcticus	0	0	0	1	0	0	0	0	0	2	0	0	0	0	0	0	0	0	0	0	0	0
Camponotus_pennsylvanicus	3	5	0	1	2	16	17	15	0	122	1	7	56	0	250	0	0	0	8	0	0	
Formica_argentea	0	0	0	0	0	78	0	0	0	6	6	0	0	0	0	0	0	0	0	0	0	0
Formica_fusca	0	0	0	20	0	1	5	0	0	4	0	0	0	0	0	0	0	0	0	0	1	0
Formica_glacialis	0	0	0	0	0	0	0	0	0	0	0	0	0	0	0	0	0	0	0	0	0	3
Formica_neogagates	0	0	0	0	0	0	0	0	0	13	5	0	0	0	0	0	0	0	0	0	0	0
Formica_obscuriventris	0	0	1	0	0	0	0	0	0	0	0	0	0	0	0	0	0	0	0	0	0	0
Formica_subintegra	0	0	0	0	0	0	0	0	0	209	0	0	0	0	0	0	0	0	0	0	0	0
Formica_subsericea	0	0	0	0	0	22	0	0	0	18	4	0	0	0	0	0	0	0	0	0	0	0
Lasius_alienus	0	0	1	1	0	0	0	0	9	1	5	0	0	0	0	3	3	5	0	4	4	4
Lasius_flavus	0	0	0	0	0	1	0	0	0	0	0	0	0	0	0	0	0	0	0	0	0	0
Lasius_neoniger	0	0	0	0	0	0	0	0	0	0	11	0	0	0	0	0	0	3	0	0	0	0
Lasius_speculiventris	0	0	0	0	0	1	0	0	0	0	0	1	0	0	0	0	0	0	0	0	0	0
Lasius_umbratus	1	0	0	0	0	0	0	0	0	0	0	1	1	0	1	0	0	0	4	0	2	0
Prenolepis_imparis	0	0	0	0	0	0	0	0	0	0	1	0	0	0	0	0	0	0	0	0	0	0
Dolichoderus_plagiatus	0	0	0	1	0	0	0	0	0	0	0	0	0	0	0	0	0	0	0	0	0	0
Tapinoma_sessile	0	0	3	1	0	0	0	0	1	3	0	2	0	0	0	0	0	0	0	0	0	0
Stenamma_brevicorne	0	0	0	2	0	0	1	0	0	0	1	0	0	0	0	0	0	0	0	0	0	0
;																						

END;

BEGIN TREES;

TRANSLATE

**Table:**

6	Amblypone_pallipes,
12	Aphaenogaster_rudis,
15	Leptothorax_curvispinosus,
16	Leptothorax_ambiguus,
17	Leptothorax_longispinosus,
22	Myrmecina_americana,
25	Myrmica_cfbrevispinosus,
26	Myrmica_detrinodis,
27	Myrmica_punctiventris,
28	Myrmica_sculptilis,
29	Myrmica_smithana,
32	Stenamma_diecki,
33	Stenamma_impar,
34	Stenamma_schmitti,
41	Camponotus_herculeanus,
42	Camponotus_noveborencensis,
43	Camponotus_nearcticus,
44	Camponotus_pennsylvanicus,
48	Formica_argentea,
49	Formica_fusca,
50	Formica_glacialis,
51	Formica_neogagates,
53	Formica_obscuriventris,
54	Formica_subintegra,
55	Formica_subsericea,
58	Lasius_alienus,
59	Lasius_flavus,
60	Lasius_neoniger,
61	Lasius_speculiventris,
62	Lasius_umbratus,
64	Prenolepis_imparis,
69	Dolichoderus_plagiatus,
71	Tapinoma_sessile,
72	Stenamma_brevicorne
;	

TREE tree_1 = ((((6:0.25):0.25):0.25):0.25,(((12:0.25):0.25):0.25, ((15:0.25,16:0.25,17:0.25):0.25):0.25,((22:0.25):0.25):0.25, ((25:0.25,26:0.25,27:0.25,28:0.25,29:0.25):0.25):0.25, ((32:0.25,33:0.25,34:0.25,72:0.25):0.25):0.25):0.25, (((41:0.25,42:0.25,43:0.25,44:0.25):0.25):0.25, ((48:0.25,49:0.25,50:0.25,51:0.25,53:0.25,54:0.25,55:0.25):0.25):0.25, ((58:0.25,59:0.25,60:0.25,61:0.25,62:0.25):0.25,(64:0.25):0.25):0.25):0.25, (((69:0.25):0.25,(71:0.25):0.25):0.25):0.25); END;

#NEXUS

[! Created by TreeMakerB v1.1.1 31/5/05]

[Species list taken from Daugherty et al 1994, distributions were simulated]

[The systematic levels used were order, suborder, infraorder, family, genus and species].

BEGIN DATA;

DIMENSIONS NTAX=62 NCHAR=15;

FORMAT DATATYPE=CONTINUOUS;

CHARSTATELABELS

**Table:**

1	site_1,
2	site_2,
3	site_3,
4	site_4,
5	site_5,
6	site_6,
7	site_7,
8	site_8,
9	site_9,
10	site_10,
11	site_11,
12	site_12,
13	site_13,
14	site_14,
15	site_15
;	

MATRIX

**Table:**

Sphenodon_guntheri	0	0	0	0	1	0	0	0	1	0	0	0	0	0	1
Sphenodon_punctulatus	1	0	0	0	0	1	0	0	0	0	1	0	0	0	0
Hoplodactylus_spPKI	0	0	0	0	0	0	0	0	1	0	0	0	1	0	1
Cyclodina_spTwo	0	0	0	1	1	0	0	0	0	0	0	0	1	0	0
Naultinus_tuberculatus	0	0	0	0	0	1	0	0	0	1	0	0	0	1	0
Hoplodactylus_chrysosireticus	1	0	0	0	0	1	0	0	0	0	1	0	0	0	0
Hoplodactylus_duvaucelii	0	0	0	0	0	0	0	1	1	1	0	0	0	0	0
Hoplodactylus_granulatus	1	1	0	0	0	0	0	0	0	0	1	0	0	0	0
Hoplodactylus_kahutarae	0	0	0	0	1	0	0	0	0	0	0	0	0	1	1
Hoplodactylus_maculatus	1	0	0	0	0	0	0	1	0	0	0	0	0	1	0
Hoplodactylus_nebulosus	0	0	0	1	0	0	1	0	0	0	1	0	0	0	0
Hoplodactylus_pacificus	1	0	0	0	1	0	0	0	0	0	0	1	0	0	0
Hoplodactylus_stephensi	0	1	0	0	0	0	0	0	0	1	0	0	0	0	1
Hoplodactylus_spNK	0	0	0	0	0	0	0	1	0	1	1	0	0	0	0
Hoplodactylus_spMa	0	0	1	0	1	0	1	0	0	0	0	0	0	0	0
Hoplodactylus_spMtA	1	0	0	1	1	0	0	0	0	0	0	0	0	0	0
Hoplodactylus_spCan	0	0	0	0	0	1	0	0	1	0	0	0	1	0	0
Hoplodactylus_spSoA	0	0	0	0	0	1	0	0	0	0	1	0	0	0	1
Hoplodactylus_spDap	0	1	1	0	0	0	0	0	0	0	1	0	0	0	0
Hoplodactylus_spEaO	1	0	0	0	1	0	0	0	0	0	0	1	0	0	0
Hoplodactylus_spWeO	0	1	0	1	0	0	0	0	1	0	0	0	0	0	0
Hoplodactylus_spSoM	0	0	0	0	1	0	0	0	0	1	0	0	0	1	0
Hoplodactylus_spWes	0	0	0	0	0	0	1	0	0	0	0	0	1	0	1
Hoplodactylus_sp3KI	0	0	0	0	0	0	1	0	0	0	0	0	0	1	1
Hoplodactylus_spMaI	0	0	1	0	1	1	0	0	0	0	0	0	0	0	0
Hoplodactylus_rakiurae	1	0	0	0	0	0	1	0	0	0	0	0	0	0	1
Naultinus_elegans	1	0	1	0	0	1	0	0	0	0	0	0	0	0	0
Naultinus_gemmeus	1	0	0	0	0	0	0	0	0	0	0	0	0	1	1
Naultinus_grayii	1	0	0	0	0	0	0	1	0	0	0	1	0	0	0
Naultinus_manukanus	0	1	0	0	0	1	0	0	0	0	1	0	0	0	0
Naultinus_rudis	0	0	1	0	0	0	0	0	1	0	0	0	0	0	1
Naultinus_stellatus	0	0	0	0	0	0	1	1	0	0	0	0	0	1	0
Cyclodina_aenea	0	0	1	0	1	0	0	0	0	0	0	0	1	0	0
Cyclodina_alani	0	0	0	1	0	0	0	0	0	1	0	0	1	0	0
Cyclodina_macgregori	1	0	0	0	0	0	0	1	1	0	0	0	0	0	0
Cyclodina_oliveri	1	0	0	0	0	0	0	0	0	1	0	0	1	0	0
Cyclodina_ornata	0	0	0	0	0	1	0	0	0	0	1	0	0	0	1
Cyclodina_whitakeri	0	1	0	0	0	0	0	0	0	1	0	0	0	1	0
Cyclodina_spOne	0	0	1	0	0	0	0	1	0	0	0	0	0	1	0
Leiolopisma_acrinasum	0	0	0	0	1	0	0	0	0	1	1	0	0	0	0
Leiolopisma_chloronoton	1	0	0	0	0	0	0	1	0	0	0	0	0	0	1
Leiolopisma_fallai	1	0	0	0	0	0	0	0	0	1	0	1	0	0	0
Leiolopisma_grande	0	1	0	0	1	1	0	0	0	0	0	0	0	0	0
Leiolopisma_homalanotum	0	0	0	0	0	0	0	0	1	0	0	0	1	0	1
Leiolopisma_inconspicuum	0	0	0	0	1	1	0	0	0	0	0	0	0	0	1
Leiolopisma_infrapunctatum	1	0	0	0	0	0	0	1	0	0	0	1	0	0	0
Leiolopisma_lineoocellatum	1	1	0	0	0	0	0	0	1	0	0	0	0	0	0
Leiolopisma_maccanni	0	0	0	1	1	1	0	0	0	0	0	0	0	0	0
Leiolopisma_microlepis	0	0	0	0	1	1	0	1	0	0	0	0	0	0	0
Leiolopisma_moco	0	1	0	0	0	1	0	0	0	0	0	0	0	1	0
Leiolopisma_nigriplantare	1	0	0	0	0	0	0	0	1	0	0	0	0	1	0
Leiolopisma_polychroma	0	0	0	0	1	1	0	0	0	0	0	0	0	0	1
Leiolopisma_notosaurus	0	0	0	0	0	0	1	1	0	0	0	0	0	0	1
Leiolopisma_otagense	0	1	0	0	0	1	0	0	0	0	1	0	0	0	0
Leiolopisma_waimatense	0	0	1	0	0	1	0	0	0	0	0	1	0	0	0
Leiolopisma_smithi	0	1	1	0	0	0	1	0	0	0	0	0	0	0	0
Leiolopisma_stenotis	0	0	0	0	1	1	0	1	0	0	0	0	0	0	0
Leiolopisma_striatum	0	0	0	0	0	1	0	1	1	0	0	0	0	0	0
Leiolopisma_suteri	0	0	0	1	0	0	1	0	0	0	0	0	1	0	0
Leiolopisma_zelandicum	1	1	0	0	0	0	0	1	0	0	0	0	0	0	0
Leiolopisma_spOne	1	0	1	0	0	0	0	0	0	0	0	1	0	0	0
Leiolopisma_spTwo	1	0	0	0	1	0	0	0	0	1	0	0	0	0	0
;															

END;

BEGIN TREES;

TRANSLATE

**Table:**

9	Sphenodon_guntheri,
8	Sphenodon_punctulatus,
40	Hoplodactylus_spPKI,
55	Cyclodina_spTwo,
47	Naultinus_tuberculatus,
19	Hoplodactylus_chrysosireticus,
20	Hoplodactylus_duvaucelii,
21	Hoplodactylus_granulatus,
22	Hoplodactylus_kahutarae,
23	Hoplodactylus_maculatus,
24	Hoplodactylus_nebulosus,
25	Hoplodactylus_pacificus,
26	Hoplodactylus_stephensi,
27	Hoplodactylus_spNK,
28	Hoplodactylus_spMa,
29	Hoplodactylus_spMtA,
30	Hoplodactylus_spCan,
31	Hoplodactylus_spSoA,
32	Hoplodactylus_spDap,
33	Hoplodactylus_spEaO,
34	Hoplodactylus_spWeO,
35	Hoplodactylus_spSoM,
36	Hoplodactylus_spWes,
37	Hoplodactylus_sp3KI,
38	Hoplodactylus_spMaI,
39	Hoplodactylus_rakiurae,
41	Naultinus_elegans,
42	Naultinus_gemmeus,
43	Naultinus_grayii,
44	Naultinus_manukanus,
45	Naultinus_rudis,
46	Naultinus_stellatus,
48	Cyclodina_aenea,
49	Cyclodina_alani,
50	Cyclodina_macgregori,
51	Cyclodina_oliveri,
52	Cyclodina_ornata,
53	Cyclodina_whitakeri,
54	Cyclodina_spOne,
56	Leiolopisma_acrinasum,
57	Leiolopisma_chloronoton,
58	Leiolopisma_fallai,
59	Leiolopisma_grande,
60	Leiolopisma_homalanotum,
61	Leiolopisma_inconspicuum,
62	Leiolopisma_infrapunctatum,
63	Leiolopisma_lineoocellatum,
64	Leiolopisma_maccanni,
65	Leiolopisma_microlepis,
66	Leiolopisma_moco,
67	Leiolopisma_nigriplantare,
68	Leiolopisma_polychroma,
69	Leiolopisma_notosaurus,
70	Leiolopisma_otagense,
71	Leiolopisma_waimatense,
72	Leiolopisma_smithi,
73	Leiolopisma_stenotis,
74	Leiolopisma_striatum,
75	Leiolopisma_suteri,
76	Leiolopisma_zelandicum,
77	Leiolopisma_spOne,
78	Leiolopisma_spTwo
;	

TREE tree_1 = ((((((8:0.1666667,9:0.1666667):0.1666667):0.1666667):0.1666667):0.1666667):0.1666667, (((((19:0.1666667,20:0.1666667,21:0.1666667,22:0.1666667,23:0.1666667,24:0.1666667,25:0.1 666667,26:0.1666667,27:0.1666667,28:0.1666667,29:0.1666667,30:0.1666667,31:0.1666667,32:0.1666667,33:0.1666667,34:0.1666667,35:0.1666667,36:0.1666667,37:0.1666667,38:0.1666667,39:0.1666667,40:0.1666667):0.1666667, (41:0.1666667,42:0.1666667,43:0.1666667,44:0.1666667,45:0.1666667,46:0.1666667,47:0.1666667):0.1666667):0.1666667):0.1666667, (((48:0.1666667,49:0.1666667,50:0.1666667,51:0.1666667,52:0.1666667,53:0.1666667,54:0.1666667,55:0.1666667):0.1666667, (56:0.1666667,57:0.1666667,58:0.1666667,59:0.1666667,60:0.1666667,61:0.1666667,62:0.1666667,63:0.1666667,64:0.1666667,65:0.1666667,66:0.1666667,67:0.1666667,68:0.1666667,69:0.1666667,70:0.1666667,71:0.1666667,72:0.1666667,73:0.1666667,74:0.1666667,75:0.1666667,76:0.1666667,77:0.1666667,78:0.1666667):0.1666667):0.1666667):0.1666667):0.1666667):0.1666667);

END;
